# Supplementary material for: Pseudomonas donghuensis HYS gtrA/B/II Gene Cluster Contributes to Its Pathogenicity toward Caenorhabditis elegans
Source: Int J Mol Sci. 2021 Oct 4;22(19):10741. doi: 10.3390/ijms221910741 (PMC8509367; doi:10.3390/ijms221910741)
Supplement: Supplementary file 1 [file ijms-22-10741-s001.zip › ijms-1370403-supplementary.pdf]

Supplementary Materials

Yaqian Xiao[1], Panning Wang[1], Xuesi Zhu[1], Zhixiong Xie[1]*

[1] Hubei Key Laboratory of Cell Homeostasis, College of Life Sciences, Wuhan University, Wuhan 430072, China

* Correspondence: zxxie@whu.edu.cn

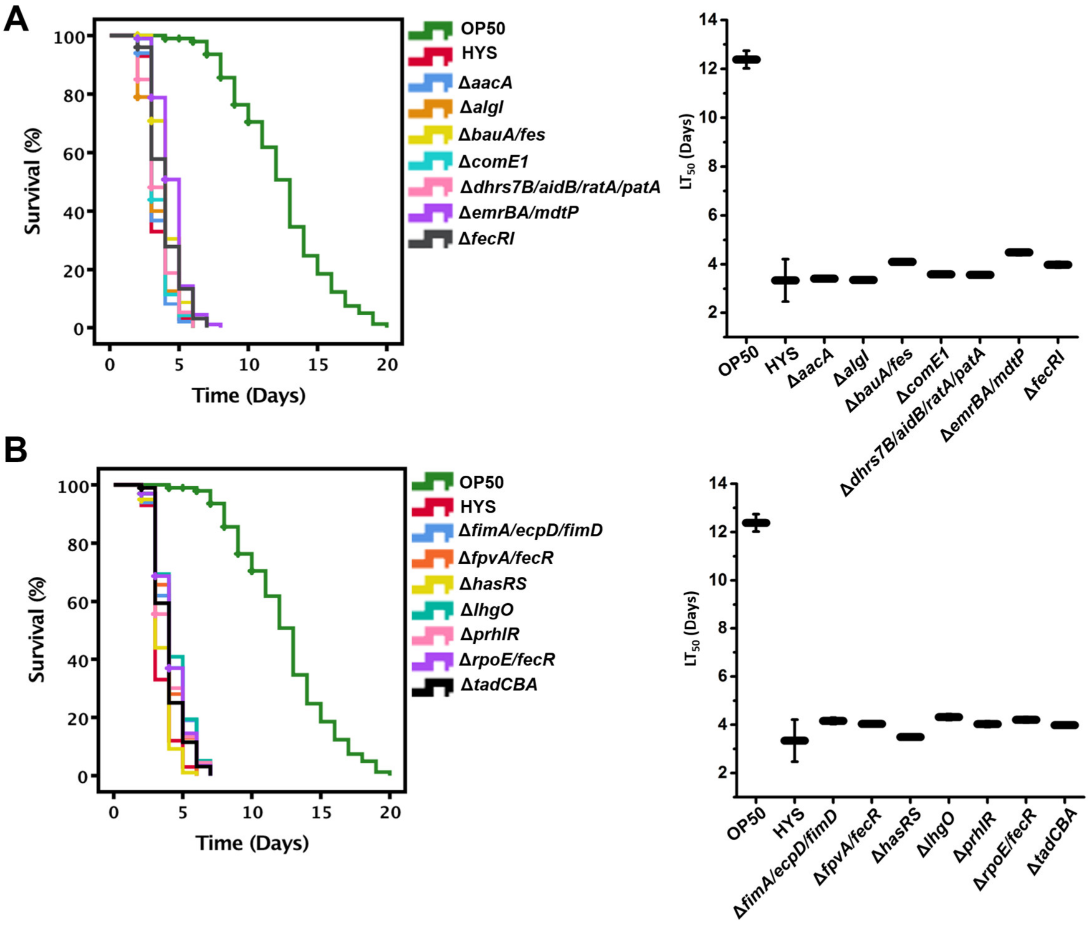

Figure S1. The functions of specific virulence genes were assessed by slow-killing experiments. (**A**,**B**) other knockout strains without significant virulence reduction were listed. *ΔmymA/mbtJ/alkK* and *ΔwzzB/rfbC/gnaT/fdtB/wzxE/asnB* have obvious growth defects, they cannot be used in the slow-killing experiments of the effect of gene virulence function loss on the life span of *C. elegans*, so they are not listed. Data are presented as the mean ± standard deviation from three independent experiments.

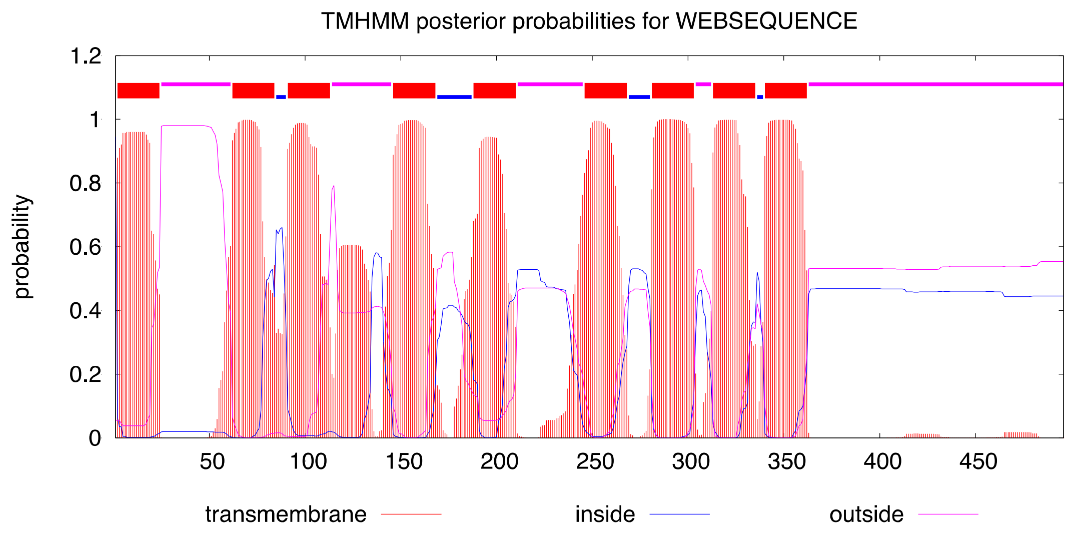

Figure S2. Hydropathy analysis of GtrII was determined using the computer program TMHMM. Red regions indicate hypothetical transmembranes with the probability for each entered on the *Y* axis. Extracellular and intracellular domains are indicated in pink and red, respectively.

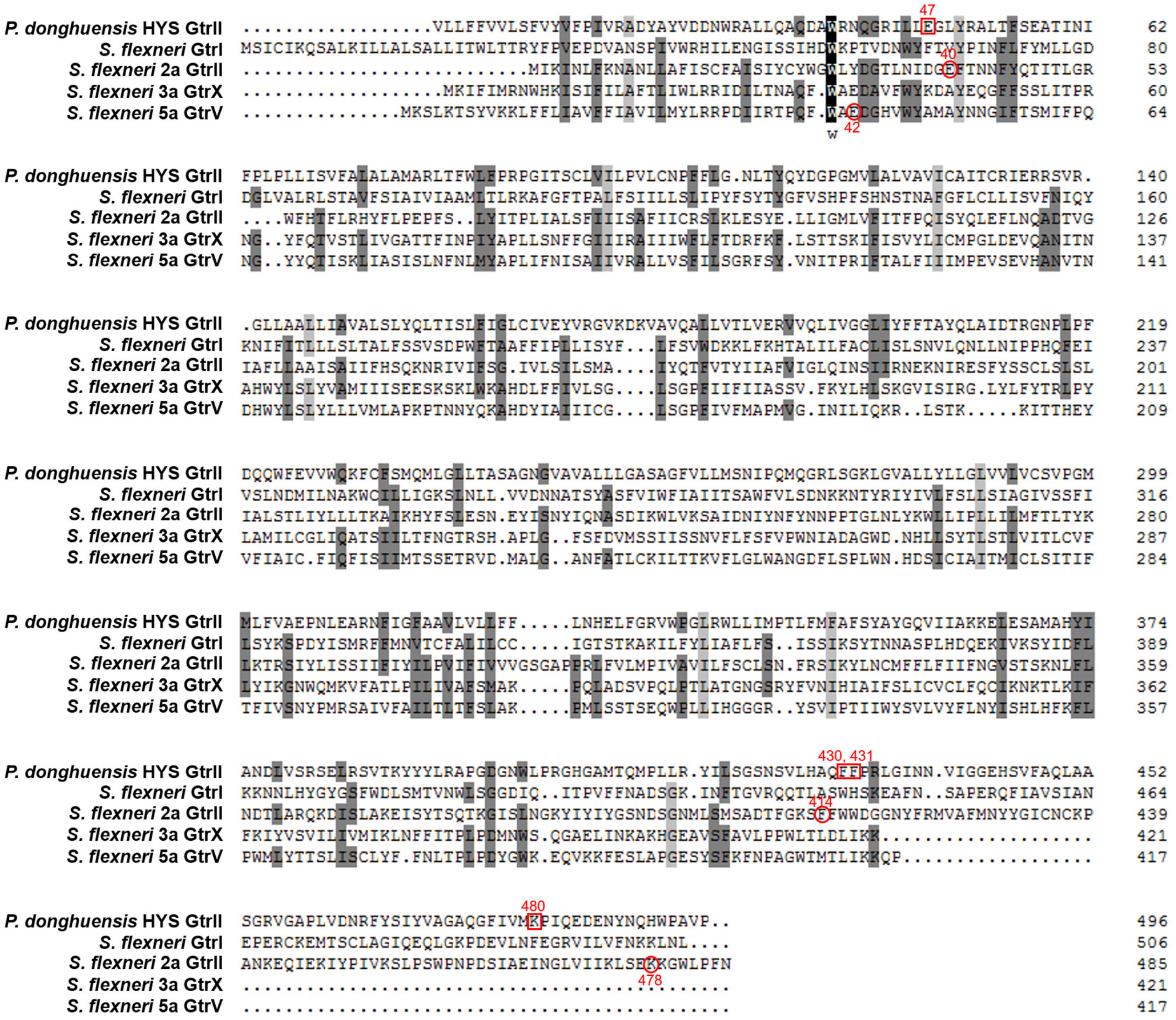

Figure S3. Sequence alignment of Gtr[type] proteins and highlight the selected residues. Protein sequences for alignment are as follows: *P. donghuensis* HYS GtrII (WP_081492764.1), *S. flexneri* GtrI (AAF09027.1), *S. flexneri* 2a GtrII (ARS43275.1), *S. flexneri* 3a GtrX (ARS43311.1) and *S. flexneri* 5a GtrV (EID60858.1). The red square indicates the critical residues in this study, each residue was converted into alanine by point mutation. The red circle means that conserved residues have been identified in *Shigella flexneri*. The number represents the position of the residue in the sequence.

Table S1. The specific virulence genes of *P. donghuensis* HYS compare to the other six strains

Gene ID	Name	Identity (VFDB)	Description
UW3_RS0105435	*fha1*	72.38	hypothetical protein
UW3_RS0117890	*fdtB*	71.35	hypothetical protein
UW3_RS26700	*fpvA*	68.02	ferrichrome-iron receptor
UW3_RS0104075	*gtrA*	42.37	bactoprenol-linked glucose translocase
UW3_RS0104080	*gtrB*	62.58	bactoprenol glucosyl transferase
UW3_RS0109785	*pilT*	61.65	twitching motility protein
UW3_RS0120465	*mymA*	61.36	putative monooxygenase
UW3_RS0105460	*impM*	60.75	hypothetical protein
UW3_RS0105440	*vasD*	56.97	lipoprotein, putative
UW3_RS0125350	*fpvA*	56.92	outer membrane ferripyoverdine receptor
UW3_RS0114930	*lhgO*	55.47	hypothetical protein
UW3_RS0117895	*ganT*	54.37	hypothetical protein
UW3_RS0124175	*hasS*	53.55	sigma factor regulatory protein FecR/PupR family
UW3_RS0117885	*wzxE*	53.55	AraC-type DNA-binding domain-containing protein
UW3_RS0117865	*asnB*	50.85	ORF_10; similar to Asparagine synthase
UW3_RS0124165	*hasR*	50.17	heme transport protein
UW3_RS0118700	*hmuU*	49.68	hemin transport system permease HmuU
UW3_RS0123355	*tadA*	48.87	type II/IV secretion system ATP hydrolase TadA
UW3_RS0102635	*irgA*	48.64	enterobactin receptor
UW3_RS0108425	*hemN*	48.05	oxygen-independent coproporphyrinogen III oxidase
UW3_RS0108420	*hemN*	42.93	oxygen-independent coproporphyrinogen III oxidase
UW3_RS0121460	*prhI*	47.44	RNA polymerase sigma factor
UW3_RS0121345	*emrA*	47.17	efflux pump protein, fatty acid resistance
UW3_RS27845	*pilM*	47.06	type IV pili biogenesis protein PilM
UW3_RS0108265	*algI*	46.84	membrane bound O-acyl transferase
UW3_RS0108825	*fecI*	46.39	heme uptake regulator
UW3_RS0117820	*comE1*	45.9	hypothetical protein
UW3_RS0111045	*aacA*	45.75	isochorismatase family protein
UW3_RS0117905	*rmlC*	45.57	dTDP-6-deoxy-D-xylo-4-hexulose-3,5-epimerase
UW3_RS0121340	*emrB*	45.45	multidrug resistance translocase
UW3_RS0113110	*fpvA*	44.91	TonB-dependent siderophore receptor
UW3_RS0110865	*pupA*	44.35	Ferric-pseudobactin 358 receptor
UW3_RS0117915	*wzzB*	43.81	Wzz [LPS O-antigen (P. aeruginosa)
UW3_RS27490	*pilR*	43.6	putative two-component system, response regulator
UW3_RS0103765	*bauA*	42.88	TonB-dependent siderophore receptor BauA
UW3_RS0121360	*pvcC*	42.36	paerucumarin biosynthesis protein PvcC

Gene ID	Name	Identity (VFDB)	Description
UW3_RS27290	*hmuV*	42.17	Hemin import ATP-binding protein HmuV
UW3_RS26865	*bvgS*	41.9	virulence sensor protein
UW3_RS0115940	*pvcA*	41.67	paerucumarin biosynthesis protein PvcA
UW3_RS0122980	*fimD*	41.4	outer membrane usher protein
UW3_RS0122975	*ecpD*	41.36	putative chaperone protein EcpD
UW3_RS0116405	*rpoE*	40.72	RNA polymerase sigma-70 factor

Table S2. Mutants of 42 specific virulence genes and their adjacent genes used in this study

Strains	Description	Gene ID	Name	Source
HYS1	Δ*aacA*	UW3_RS0111045	*aacA*	This study
HYS2	Δ*algI*	UW3_RS0108265	*algI*	This study
HYS3	Δ*bauA/fes*	UW3_RS0103765<br>UW3_RS0103770	*bauA*<br>*fes*	This study
HYS4	Δ*comE1*	UW3_RS0117820	*comE1*	This study
HYS5	Δ*dhrs7B/aidB/ratA/patA*	UW3_RS0118235<br>UW3_RS0118230<br>UW3_RS0118225<br>UW3_RS0118220	*dhrs7B*<br>*aidB*<br>*ratA*<br>*patA*	This study
HYS6	Δ*emrBA/mdtP*	UW3_RS0121340<br>UW3_RS0121345<br>UW3_RS27465	*emrB*<br>*emrA*<br>*mdtP*	This study
HYS7	Δ*fecRI*	UW3_RS0108830<br>UW3_RS0108825	*fecR*<br>*fecI*	This study
HYS8	Δ*fimA/ecpD/fimD*	UW3_RS0122970<br>UW3_RS0122975<br>UW3_RS0122980	*fimA*<br>*ecpD*<br>*fimD*	This study
HYS9	Δ*fpvA/fecR*	UW3_RS0125350<br>UW3_RS0125355	*fpvA*<br>*fecR*	This study
HYS10	Δ*gtrAB*	UW3_RS0104075<br>UW3_RS0104080	*gtrA*<br>*gtrB*	This study
HYS11	Δ*hasRS*	UW3_RS0124165<br>UW3_RS0124175	*hasR*<br>*hasS*	This study
HYS12	Δ*hemN*/hem*N*	UW3_RS0108425<br>UW3_RS0108420	*hemN*<br>*hemN*	This study
HYS13	Δ*hurR/hmuVUTS*	UW3_RS0118690<br>UW3_RS27290<br>UW3_RS0118700	*hurR*<br>*hmuV*<br>*hmuU*	This study

Strains	Description	Gene ID	Name	Source
		UW3_RS0118705	*hmuT*	
		UW3_RS0118710	*hmuS*	
HYS14	Δ*irgA*	UW3_RS0102635	*irgA*	This study
HYS15	Δ*lhgO*	UW3_RS0114930	*lhgO*	This study
HYS16	Δ*mymA*/*mbtJ*/*alkK*	UW3_RS0120455	*mbtJ*	This study
		UW3_RS0120460	*alkK*	
		UW3_RS0120465	*mymA*	
HYS17	Δ*prhIR*	UW3_RS0121460	*prhI*	This study
		UW3_RS0121455	*prhR*	
HYS18	Δ*rpoE*/*fecR*	UW3_RS0116405	*rpoE*	This study
		UW3_RS0116400	*fecR*	
HYS19	Δ*tadCBA*	UW3_RS0123365	*tadC*	This study
		UW3_RS0123360	*tadB*	
		UW3_RS0123355	*tadA*	
HYS20	Δ*wzzB*/*rfbC*/*gnaT*/ *fdtB*/*wzxE*/*asnB*	UW3_RS0117915	*wzzB*	This study
		UW3_RS0117905	*rfbC*	
		UW3_RS0117895	*gnaT*	
		UW3_RS0117890	*fdtB*	
		UW3_RS0124175	*wzxE*	
		UW3_RS0117865	*asnB*	

Table S3. Bacterial strains and plasmids used in this study

Strains and Plasmids	Description[a]	Source
E. coli strain		
S17-1 λpir	*thi pro hsdR recA*; chromosomal RP4-2; (Tc::Mu) (Km::Tn7) Tp^r Sp^r	(Simon, Priefer et al. 1983)[b]
Pseudomonas donghuensis strains		
HYS	Wild-type, lethal to *C. elegans*, Cm^r	Preserved in laboratory
HYS21	Δ*gtrA*	This study
HYS22	Δ*gtrB*	This study
HYS23	Δ*gtrII*	This study
HYS24	Δ*gtrAB*	This study
HYS25	Δ*gtrABII*	This study
HYS26	Δ*gtrA*/pBBR1-MCS2	This study
HYS27	Δ*gtrA*/pBBR2-*gtrA*	This study
HYS28	Δ*gtrB*/pBBR1-MCS2	This study

Strains and Plasmids	Description[a]	Source
HYS29	Δ*gtrB*/pBBR2-*gtrB*	This study
HYS30	Δ*gtrII*/pBBR1-MCS2	This study
HYS31	Δ*gtrII*/pBBR2-*gtrII*	This study
HYS32	Δ*gtrABII*/pBBR1-MCS2	This study
HYS33	Δ*gtrABII*/pBBR2-*gtrA*	This study
HYS34	Δ*gtrABII*/pBBR2-*gtrB*	This study
HYS35	Δ*gtrABII*/pBBR2-*gtrII*	This study
HYS36	Δ*gtrABII*/pBBR2-*gtrABII*	This study
HYS37	HYS/pBBR1-MCS2	This study
Plasmids		
pEX18Gm	Gene replacement vector, Gm^r *oriT*$^+$ *sacB*$^+$	(Hoang, Karkhoff-Schweizer et al. 1998)[c]
pEX18Gm-*gtrA*-UD	Gene replacement vector for *gtrA*	This study
pEX18Gm-*gtrB*-UD	Gene replacement vector for *gtrB*	This study
pEX18Gm-*gtrII*-UD	Gene replacement vector for *gtrII*	This study
pEX18Gm-*gtrAB*-UD	Gene replacement vector for *gtrAB*	This study
pEX18Gm-*gtrABII*-UD	Gene replacement vector for *gtrABII*	This study
pBBR1-MCS2	Mobilizable broad-host-range cloning vector, Km^r	(Kovach, Elzer et al. 1995)[d]
pBBR2-*gtrA*	Cloning vector for *gtrA*	This study
pBBR2-*gtrB*	Cloning vector for *gtrB*	This study
pBBR2-*gtrII*	Cloning vector for *gtrII*	This study
pBBR2-*gtrABII*	Cloning vector for *gtrABII*	This study

[a] Cm, chloramphenicol, Gm, gentamicin, Km, kanamycin; [b] Bio-Technology 1(9): 784-791; [c] Gene 212(1): 77-86; [d] Gene 166(1): 175-176.

Table S4. Oligonucleotide primers used in this study

Primer	Sequence (5′-3′)[a]	Description
aacA-up-1	CGGGATCCAGACCACTGCCTGAAACC	Construction of Δ*aacA*
aacA-up-2	GGAATTCCACCACAACCTGACCCT	Construction of Δ*aacA*
aacA-down-1	GGAATTCCATATCCAGGACCAGCAGT	Construction of Δ*aacA*
aacA-down-2	GCTCTAGATGGTTGTCTTCGGTCAGG	Construction of Δ*aacA*
aacA-M-1	GGCTTGGCTTTGACTTG	Verification of Δ*aacA*

Primer	Sequence (5′-3′)[a]	Description
aacA-M-2	CGAACGGTCGCAGGTA	Verification of Δ*aacA*
algI-up-1	CGGGATCCCCCTTGACCGTCTTGT	Construction of Δ*algI*
algI-up-2	CCCAAGCTTCGGCGCATTCGTT	Construction of Δ*algI*
algI-down-1	CCCAAGCTTCCACCAGGCATAGA	Construction of Δ*algI*
algI-down-2	GCTCTAGAGGCAAACGCTTTCACG	Construction of Δ*algI*
algI-M-1	GCTTCCACCGAAACACTG	Verification of Δ*algI*
algI-M-2	GCACGAGAACGACCACA	Verification of Δ*algI*
bauA/fes-up-1	GCTCTAGAGCGGTTGGTGGTCGTTC	Construction of Δ*bauA/fes*
bauA/fes-up-2	GGAATTCGGCGACCAAGCCAGTGTT	Construction of Δ*bauA/fes*
bauA/fes-down-1	GGAATTCGGGCAAGAAGGTGAAGAGC	Construction of Δ*bauA/fes*
bauA/fes-down-2	CGGGATCCCACGGGAGATGGAGAACG	Construction of Δ*bauA/fes*
bauA/fes-M-1	ACGGCTCGCAGGTGTTG	Verification of Δ*bauA/fes*
bauA/fes-M-2	TGGTGTACCGCTTTGACG	Verification of Δ*bauA/fes*
comE1-up-1	ACGCGTCGACGGTCAGGCTCAGGA	Construction of Δ*comE1*
comE1-up-2	GCTCTAGACAAGGCACTGCTGGA	Construction of Δ*comE1*
comE1-down-1	GCTCTAGAGATGTCGTAAGGGAT	Construction of Δ*comE1*
comE1-down-2	CGGGATCCTGAAAGATGGGCTGAC	Construction of Δ*comE1*

Primer	Sequence (5′-3′)[a]	Description
comE1-M-1	GCAGTGACGGAATGG	Verification of Δ*comE1*
comE1-M-2	GAGAATGAAGTAGAGTGGC	Verification of Δ*comE1*
dhrs7B/aidB/ratA/patA-up-1	CGGGATCCGCAGATGTGCCGCTACG	Construction of Δ*dhrs7B/aidB/ratA/pat*
dhrs7B/aidB/ratA/patA-up-2	GGAATTCCAGTTGCTCGCTGACCTT	Construction of Δ*dhrs7B/aidB/ratA/pat*
dhrs7B/aidB/ratA/patA-down-1	GGAATTCGGTCAGCCTGCCATTGTAC	Construction of Δ*dhrs7B/aidB/ratA/pat*
dhrs7B/aidB/ratA/patA-down-2	GCTCTAGAACGCCTGGAAACCCTGT	Construction of Δ*dhrs7B/aidB/ratA/pat*
dhrs7B/aidB/ratA/patA-M-1	CACCCACCCTTGCGACTA	Verification of Δ*dhrs7B/aidB/ratA/pat*
dhrs7B/aidB/ratA/patA-M-2	TTGAAAGCCTGCTGATGC	Verification of Δ*dhrs7B/aidB/ratA/pat*
emrBA/mdtP-up-1	GCTCTAGAGGCTTCCAGCACCACCA	Construction of Δ*emrBA/mdtP*
emrBA/mdtP-up-2	GGAATTCTGACGCCGTTCGACCAC	Construction of Δ*emrBA/mdtP*
emrBA/mdtP-down-1	GGAATTCATGCCAGCAGCAGACAGA	Construction of Δ*emrBA/mdtP*
emrBA/mdtP-down-2	CGGGATCCACCACGCCGACAAAGG	Construction of Δ*emrBA/mdtP*
emrBA/mdtP-M-1	TCGAGACAGTGGGTGGG	Verification of Δ*emrBA/mdtP*
emrBA/mdtP-M-2	CAGCGGTAGCGGGAAA	Verification of Δ*emrBA/mdtP*
fecRI-up-1	CGGATCCAACAGCTTGAGGCGGTAA	Construction of Δ*fecRI*
fecRI-up-2	GGAATTCCATCGTGCGGGCAGTT	Construction of Δ*fecRI*
fecRI-down-1	GGAATTCAGCGAGCGTACCTTG	Construction of Δ*fecRI*

Primer	Sequence (5′-3′)[a]	Description
fecRI-down-2	GCTCTAGACCACATAGCCCTGAACC	Construction of Δ*fecRI*
fecRI-M-1	GCGGCGATGTCAGTTGG	Verification of Δ*fecRI*
fecRI-M-2	CGAGGTGTCGGTCTTGGTC	Verification of Δ*fecRI*
fimA/*ecpD*/*fimD*-up-1	GCTCTAGAGGATAAGTGGGCTGAAAC	Construction of Δ*fimA*/*ecpD*/*fimD*
fimA/*ecpD*/*fimD* -up-2	GGAATTCTGGGCATGGAGACTGATA	Construction of Δ*fimA*/*ecpD*/*fimD*
fimA/*ecpD*/*fimD* -down-1	GGAATTCTCGGACAAGGCAGCCAACT	Construction of Δ*fimA*/*ecpD*/*fimD*
fimA/*ecpD*/*fimD* -down-2	GCTCTAGATGCCCACGGCGTCAAA	Construction of Δ*fimA*/*ecpD*/*fimD*
fimA/*ecpD*/*fimD* -M-1	CAACTGGGCGATGTAATGC	Verification of Δ*fimA*/*ecpD*/*fimD*
fimA/*ecpD*/*fimD* -M-2	TGCTGGCGTCGGTGATG	Verification of Δ*fimA*/*ecpD*/*fimD*
fpvA/*fecR*-up-1	CGGGATCCACCGCCATCACATAGAACG	Construction of Δ*fpvA*/*fecR*
fpvA/*fecR* -up-2	GGAATTCGTCAACGGCAACAACCTG	Construction of Δ*fpvA*/*fecR*
fpvA/*fecR* -down-1	GGAATTCTCGGCTTCGCTACGCT	Construction of Δ*fpvA*/*fecR*
fpvA/*fecR* -down-2	GCTCTAGATGCTGATTCTGGAGACCCT	Construction of Δ*fpvA*/*fecR*
fpvA/*fecR* -M-1	CGCTGAGCATTGCCACGAA	Verification of Δ*fpvA*/*fecR*
fpvA/*fecR* -M-2	TACGCATCCTCGCCTCCTCC	Verification of Δ*fpvA*/*fecR*
hasRS-up-1	CGGGATCCGCACCGTGGGTCGCATACT	Construction of Δ*hasRS*
hasRS-up-2	CCCAAGCTTCCCACAACTACTCCTACCTC	Construction of Δ*hasRS*

Primer	Sequence (5′-3′)[a]	Description
hasRS-down-1	CCCAAGCTTAAGCGAGCCAAGACGG	Construction of Δ*hasRS*
hasRS-down-2	GCTCTAGACGCCACTGAACAGCACAT	Construction of Δ*hasRS*
hasRS-M-1	GAGCGGGTTGCTTTCAT	Verification of Δ*hasRS*
hasRS-M-2	CCATCCACAGGGAGTTCG	Verification of Δ*hasRS*
hemN/*hemN*-up-1	GCTCTAGAAGCACATCCGCAAACAG	Construction of Δ*hemN*/*hemN*
hemN/*hemN*-up-2	GGAATTCCAGGCAGTGCAGATAGCG	Construction of Δ*hemN*/*hemN*
hemN/*hemN*-down-1	GGAATTCTGGCATCCGCTTCAACG	Construction of Δ*hemN*/*hemN*
hemN/*hemN*-down-2	CGGGATCCTCTCGACGACTTCTCCTTCT	Construction of Δ*hemN*/*hemN*
hemN/*hemN*-M-1	CTGCGGGAAGATGAACC	Verification of Δ*hemN*/*hemN*
hemN/*hemN*-M-2	CCAGTACGACCTGCACAAC	Verification of Δ*hemN*/*hemN*
hurR/*hmuVUTS*-up-1	GCTCTAGAGGACCTGTTCAGCATTCG	Construction of Δ*hurR*/*hmuVUTS*
hurR/*hmuVUTS*-up-2	GGAATTCCGGTGAGCCATTGTGC	Construction of Δ*hurR*/*hmuVUTS*
hurR/*hmuVUTS*-down-1	GGAATTCCTAAGGCTGTAGACACCCACC	Construction of Δ*hurR*/*hmuVUTS*
hurR/*hmuVUTS*-down-2	CGCGGATCCCCCGGTGAATGAAG	Construction of Δ*hurR*/*hmuVUTS*
hurR/*hmuVUTS*-M-1	GCACCGTCATCACCCA	Verification of Δ*hurR*/*hmuVUTS*
hurR/*hmuVUTS*-M-2	GACCTCGTTCGGCTTC	Verification of Δ*hurR*/*hmuVUTS*
irgA-up-1	CGGGATCCTCCTGACGGCGGTAGTGA	Construction of Δ*irgA*

Primer	Sequence (5′-3′)[a]	Description
irgA-up-2	GGAATTCTCACGGTAGGACTTGTT	Construction of Δ*irgA*
irgA-down-1	GGAATTCCCTATCGGGTGACCAAG	Construction of Δ*irgA*
irgA-down-2	GCTCTAGAATCGCAAACAGCAACC	Construction of Δ*irgA*
irgA-M-1	TGCCGACACCGAGGAT	Verification of Δ*irgA*
irgA-M-2	GGAAGTGGAAGCGATAGAAG	Verification of Δ*irgA*
lhgO-up-1	GCTCTAGAGGCGTGCTCGTAGGAGGTCA	Construction of Δ*lhgO*
lhgO-up-2	GGAATTCCCTGTTCGCCGAGACCC	Construction of Δ*lhgO*
lhgO-down-1	GGAATTCCACGCCGCTGTTATGC	Construction of Δ*lhgO*
lhgO-down-2	CGGGATCCGATTACTTCGTCGCCTT	Construction of Δ*lhgO*
lhgO-M-1	GCCTTTGAGGTCGTTCC	Verification of Δ*lhgO*
lhgO-M-2	GCACGGTATTCCGCTTT	Verification of Δ*lhgO*
mymA/*mbtJ*/*alkK*-up-1	GCTCTAGATCCGATAAGGCGACCCA	Construction of Δ*mymA*/*mbtJ*/*alkK*
mymA/*mbtJ*/*alkK*-up-2	GGAATTCGCCCAGCAACAAGCACTC	Construction of Δ*mymA*/*mbtJ*/*alkK*
mymA/*mbtJ*/*alkK*-down-1	GGAATTCGCGAGATTGCGGTTGG	Construction of Δ*mymA*/*mbtJ*/*alkK*
mymA/*mbtJ*/*alkK*-down-2	CGGGATCCGGCAGATGAGCGGTTGT	Construction of Δ*mymA*/*mbtJ*/*alkK*
mymA/*mbtJ*/*alkK*-M-1	CAGGGCGTGCAACAGAG	Verification of Δ*mymA*/*mbtJ*/*alkK*
mymA/*mbtJ*/*alkK*-M-2	CATTGAGCGACGACCAGAT	Verification of Δ*mymA*/*mbtJ*/*alkK*

Primer	Sequence (5′-3′)[a]	Description
prhIR-up-1	GCTCTAGATCTGCTGGCGGGTCAT	Construction of Δ*prhIR*
prhIR-up-2	CCCAAGCTTCGGTCAAGGTCGATCAATA	Construction of Δ*prhIR*
prhIR-down-1	CCCAAGCTTGCAGGGCCGACAAACGT	Construction of Δ*prhIR*
prhIR-down-2	CGGGATCCGAACGTCTGGTGGGTCAA	Construction of Δ*prhIR*
prhIR-M-1	TCCTCGTCCGCCACAA	Verification of Δ*prhIR*
prhIR-M-2	GACAGCAACGCAAACATCA	Verification of Δ*prhIR*
rpoE/fecR-up-1	CGGGATCCAGGAGCCACTGTCTTCGG	Construction of Δ*rpoE/fecR*
rpoE/fecR-up-2	GGAATTCTTCGATGCCAAGGCTCT	Construction of Δ*rpoE/fecR*
rpoE/fecR-down-1	GGAATTCAGCAGGAGGAGGCACTT	Construction of Δ*rpoE/fecR*
rpoE/fecR-down-2	GCTCTAGAGGCGTCCTGGATGATTT	Construction of Δ*rpoE/fecR*
rpoE/fecR-M-1	TGGCGTTGTCGTTGAGTG	Verification of Δ*rpoE/fecR*
rpoE/fecR-M-2	ATCCCTGTCCGTTGTCTGT	Verification of Δ*rpoE/fecR*
tadCBA-up-1	GCTCTAGAGGTGATGAATGCCAAGGG	Construction of Δ*tadCBA*
tadCBA-up-2	CCCAAGCTTTTCTCCCGCCAGTCGGTCTC	Construction of Δ*tadCBA*
tadCBA-down-1	CCCAAGCTTGGTATCCGAGGAAATG	Construction of Δ*tadCBA*
tadCBA-down-2	GGAATTCGCTTGCCCTCCTCTGCTT	Construction of Δ*tadCBA*
tadCBA-M-1	AGAGCGACCGCCATTG	Verification of Δ*tadCBA*

Primer	Sequence (5′-3′)[a]	Description
tadCBA-M-2	GGCAGAGGCCCCTTGT	Verification of Δ*tadCBA*
wzzB/*rfbC*/*gnaT*/ *fdtB*/*wzxE*/*asnB*-up-1	GCTCTAGATCTCGCTGTCAAACTCC	Construction of Δ*wzzB*/*rfbC*/*gnaT*/ *fdtB*/*wzxE*/*asnB* -
wzzB/*rfbC*/*gnaT*/ *fdtB*/*wzxE*/*asnB*-up-2	GGAATTCAGCGGCGATTGGAG	Construction of Δ*wzzB*/*rfbC*/*gnaT*/ *fdtB*/*wzxE*/*asnB*
wzzB/*rfbC*/*gnaT*/ *fdtB*/*wzxE*/*asnB*-down-1	GGAATTCGAGGGAAGGTAGTGCG	Construction of Δ*wzzB*/*rfbC*/*gnaT*/ *fdtB*/*wzxE*/*asnB*
wzzB/*rfbC*/*gnaT*/ *fdtB*/*wzxE*/*asnBB*-down-2	CGGGATCCTGGCGTTCTACTTCACTC	Construction of Δ*wzzB*/*rfbC*/*gnaT*/ *fdtB*/*wzxE*/*asnB*
wzzB/*rfbC*/*gnaT*/ *fdtB*/*wzxE*/*asnB*-M-1	TCGCCAAGTCTGCC	Verification of Δ*wzzB*/*rfbC*/*gnaT*/ *fdtB*/*wzxE*/*asnB*
wzzB/*rfbC*/*gnaT*/ *fdtB*/*wzxE*/*asnB*-M-2	CCGCCGTGATGTTT	Verification of Δ*wzzB*/*rfbC*/*gnaT*/ *fdtB*/*wzxE*/*asnB*
gtrA-up-1	CGGGATCCCGGCGAGAAATAC	Construction of Δ*gtrA*
gtrA-up-2	GGAATTCCGATCACGGCATAGGT	Construction of Δ*gtrA*
gtrA-down-1	CGGAATTCCTGACCTGCGGCTT	Construction of Δ*gtrA*
gtrA-down-2	GCTCTAGACCTTGCACCATTGCTCGA	Construction of Δ*gtrA*
gtrA-M-1	GCCCGTTGCCCGTAAA	Verification of Δ*gtrA*
gtrA-M-2	GTCTTGATTACGTCCACCACC	Verification of Δ*gtrA*
gtrB-up-1	GCTCTAGACAGCGGGCTCTTGAA	Construction of Δ*gtrB*
gtrB-up-2	GGAATTCCACGATCAGTGAAACCT	Construction of Δ*gtrB*

Primer	Sequence (5′-3′)[a]	Description
gtrB-down-1	GGAATTCACGACCTATCGAAAGGAGACC	Construction of Δ*gtrB*
gtrB-down-2	CGGGATCCCCAGTTGGTAAGCGGTA	Construction of Δ*gtrB*
gtrB-M-1	GCCCATTTCCTCAACCAG	Verification of Δ*gtrB*
gtrB-M-2	TGATAGCCGCCCTTGC	Verification of Δ*gtrB*
gtrII-up-1	GGAATTCGCTTGGCGGCTATGGA	Construction of Δ*gtrII*
gtrII-up-2	CCCAAGCTTTCATCCACATAGGCATAATC	Construction of Δ*gtrII*
gtrII-down-1	CCCAAGCTTGCGGTGAGCATTCGGTCT	Construction of Δ*gtrII*
gtrII-down-2	GCTCTAGAAACTTCCACCACAACACCCTC	Construction of Δ*gtrII*
gtrII-M-1	GCAAGGTGGTGGACGTAATC	Verification of Δ*gtrII*
gtrII-M-2	GCAGTTCGTCGCCCAGTT	Verification of Δ*gtrII*
gtrAB-up-1	CGGGATCCAGGTTGGGTTTGAAGCG	Construction of Δ*gtrAB*
gtrAB-up-2	GGAATTCCGACGAAGGTGTAGAGGG	Construction of Δ*gtrAB*
gtrAB-down-1	GGAATTCACCTCGTTCAGCACCTT	Construction of Δ*gtrAB*
gtrAB-down-2	GCTCTAGAAGGGCAGCGGGAAA	Construction of Δ*gtrAB*
gtrAB-M-1	AGCGGTAGCGGGTCCAT	Verification of Δ*gtrAB*
gtrAB-M-2	GGTCAGGTTGCCGAGGA	Verification of Δ*gtrAB*
gtrABII-up-1	GGAATTCGCACCACATACAGCACCCA	Construction of Δ*gtrABII*

Primer	Sequence (5′-3′)[a]	Description
gtrABII-up-2	CCCAAGCTTACGACGAAGGTGTAGAGG	Construction of Δ*gtrABII*
gtrABII-down-1	CCCAAGCTTGCGGTGAGCATTCGGTCT	Construction of Δ*gtrABII*
gtrABII-down-2	GCTCTAGAAACTTCCACCACAACACCCTC	Construction of Δ*gtrABII*
gtrABII-M-1	TCAGGTTGGGTTTGAAGCG	Verification of Δ*gtrABII*
gtrABII-M-2	CTGCATCCAGTCGCAAAGC	Verification of Δ*gtrABII*
gtrA-up/EcoR	CGGGATCCAGACGAATTTCGGCTTAC	Amplification of *gtrA*
gtrA-down/XbaF	GCTCTAGATTGAACACTGGCACGAT	Amplification of *gtrA*
gtrB-up/EcoR	CGGGATCCCCTGACCTGCGGCTTCT	Amplification of *gtrB*
gtrB-down/XbaF	GCTCTAGACACGTGGCTCTGACTTAA	Amplification of *gtrB*
gtrII-up/EcoR	GGAATTCTCGGCATCGGCATAT	Amplification of *gtrII*
gtrII-down/XbaF	CGGGATCCCAGTTTCGAGCCACAGG	Amplification of *gtrII*
gtrABII-up/EcoR	GGAATTCGCTTACGTGGCCTGCTTGG	Amplification of *gtrABII*
gtrABII-down/XbaF	CGGGATCCCGGGCGGACAGTTTCGA	Amplification of *gtrABII*
gtrII(E47A)-up-1	CGGGAATTCGCTTGGCGGCTATGGA	Construction of *gtrII*(E47A)
gtrII(E47A)-up-2	CAGCGCCCGGTACAGACC**CGC**AAGCAAGA TCCTGCCTTGG	Construction of *gtrII*(E47A)
gtrII(E47A)-down-1	CCAAGGCAGGATCTTGCTT**GCG**GGTCTGTA CCGGGCGCTG	Construction of *gtrII*(E47A)
gtrII(E47A)-down-2	CGGGATCCGGACCGCAACCTTGTCTTTT	Construction of *gtrII*(E47A)

Primer	Sequence (5′-3′)[a]	Description
gtrII(E47A)-up-1	GCTCTAGAATCACGAGTTGTTTGGGCGGG	Construction of *gtrII*(F430A)
gtrII(F430A)-up-2	GATCCCCAAGCGCGGGAA**AGC**CTGAGCGT GCAACACTGAG	Construction of *gtrII*(F430A)
gtrII(F430A)-down-1	CTCAGTGTTGCACGCTCAG**GCT**TTCCCGCG CTTGGGGATC	Construction of *gtrII*(F430A)
gtrII(F430A)-down-2	CGGGATCCCGCAACTGGAAGCCAAGCA	Construction of *gtrII*(F430A)
gtrII(F431A)-up-2	CTCAGTGTTGCACGCTCAGTTT**GCC**CCGCG CTTGGGGATC	Construction of *gtrII*(F431A)
gtrII(F431A)-down-1	GATCCCCAAGCGCGG**GGC**AAACTGAGCGT GCAACACTGAG	Construction of *gtrII*(F431A)
gtrII(K480A)-up-2	TCATCTTCCTGTATGGG**CGC**CATGACAATG AAGCCCTGCG	Construction of *gtrII*(K480A)
gtrII(K480A)-down-1	CGCAGGGCTTCATTGTCATG**GCG**CCCATAC AGGAAGATGA	Construction of *gtrII*(K480A)

[a] Restriction sites are underlined.
